# Forced Expiratory Flow (FEF_25–75%_) as a Clinical Endpoint in Children and Adolescents with Symptomatic Asthma Receiving Tiotropium: A Post Hoc Analysis

**DOI:** 10.1007/s41030-020-00117-6

**Published:** 2020-05-12

**Authors:** Stanley J. Szefler, Stanley Goldstein, Christian Vogelberg, George W. Bensch, John Given, Branko Jugovic, Michael Engel, Petra M. Moroni-Zentgraf, Ralf Sigmund, Eckard H. Hamelmann

**Affiliations:** 1 Department of Pediatrics, The Breathing Institute, Children’s Hospital of Colorado, University of Colorado School of Medicine, Aurora, CO USA; 2 Allergy and Asthma Care of Long Island, Rockville Centre, NY USA; 3 University Hospital Carl Gustav Carus, Technical University of Dresden, Dresden, Germany; 4 Allergy, Immunology and Asthma Medical Group, Inc., Stockton, CA USA; 5 Allergy and Respiratory Center, Canton, OH USA; 6 TA Respiratory Diseases/Biosimilars Medicine, Boehringer Ingelheim International GmbH, Ingelheim am Rhein, Germany; 7 Boehringer Ingelheim Pty Ltd, Sydney, NSW Australia; 8 Global Biometrics and Data Sciences, Boehringer Ingelheim Pharma GmbH & Co. KG., Biberach an der Riss, Germany; 9 Klinik für Kinder- Und Jugendmedizin, Kinderzentrum Bethel, Evangelisches Klinikum Bethel EvKB, Bielefeld, Germany

**Keywords:** Airway obstruction, Asthma, Muscarinic antagonist, Tiotropium

## Abstract

**Introduction:**

In pediatric patients with asthma, measurements of forced expiratory volume in 1 s (FEV_1_) may be normal or may not correlate with symptom severity. Forced expiratory flow at 25–75% of the vital capacity (FEF_25–75%_) is a potentially more sensitive parameter for assessing peripheral airway function. This post hoc analysis compared FEF_25–75%_ with FEV_1_ as an endpoint to assess bronchodilator responsiveness in children with asthma.

**Methods:**

Change from baseline in trough FEF_25–75%_ and trough FEV_1_ following treatment with either tiotropium (5 µg or 2.5 µg) or placebo Respimat^®^ was analyzed in four phase III trials in children (aged 6–11 years) and adolescents (aged 12–17 years) with symptomatic moderate (VivaTinA-asthma^®^ and PensieTinA-asthma^®^) and mild (CanoTinA-asthma^®^ and RubaTinA-asthma^®^) asthma. Data from all treatment arms were pooled and correlations between FEF_25–75%_ and FEV_1_ were calculated and analyzed.

**Results:**

A total of 1590 patients were included in the analysis. Tiotropium Respimat^®^ consistently improved FEF_25–75%_ and FEV_1_ versus placebo, although in adolescents with severe asthma, the observed improvements were not statistically significant. Improvements in FEF_25–75%_ response with tiotropium versus placebo were largely more pronounced than improvements in FEV_1_. Statistical assessment of the correlation of FEV_1_ and FEF_25–75%_ showed moderate-to-high correlations (Pearson’s correlation coefficients 0.73–0.80).

**Conclusions:**

In pediatric patients, FEF_25–75%_ may be a more sensitive measure to detect treatment response, certainly to tiotropium, than FEV_1_ and should be evaluated as an additional lung function measurement.

**Electronic Supplementary Material:**

The online version of this article (10.1007/s41030-020-00117-6) contains supplementary material, which is available to authorized users.

## Key Summary Points

**Table:** 

Interpretation of lung function data from children and adolescents can be challenging because standard measures such as forced expiratory volume in 1 s (FEV_1_) do not always correlate with symptom severity.
Forced expiratory flow at 25–75% of the vital capacity (FEF_25–75%_) could be a more sensitive measure of peripheral airway function than FEV_1_ in these patients.
Using pooled data from four phase III trials in patients with asthma aged 6–17 years, we investigated change from baseline in trough FEF_25–75%_ and FEV_1_ following treatment with either tiotropium (5 µg or 2.5 µg) or placebo Respimat^®^. Trough was defined as the pre-dose FEF_25–75%_ or FEV_1_, respectively, measured 24 h post previous drug administration, 10 min prior to the evening dose of usual asthma medication and daily dose of randomized treatment.
Tiotropium Respimat^®^ consistently improved FEF_25–75%_ and FEV_1_ versus placebo, with improvements in FEF_25–75%_ largely more pronounced than those seen in FEV_1_. Improvements were statistically significant versus placebo except in adolescents with severe asthma.
FEF_25–75%_ may be a more sensitive measure to detect treatment response, certainly to tiotropium, than FEV_1_ and should be evaluated as an additional lung function measurement in pediatric patients.

## Commentary

Assessment of standard measures of lung function can be more challenging in children and adolescents compared with adults. Whilst forced expiratory volume in 1 s (FEV_1_) is accepted as a standard measure of lung function in adults with asthma, it is often found to be normal in pediatric patients, and measurements may not always correlate with symptom severity [1].

Forced expiratory flow at 25–75% of the vital capacity (FEF_25–75%_) is potentially a more sensitive parameter than FEV_1_ for assessing changes in peripheral airway function in pediatric patients [2, 3]. Indeed, Vilozni et al. reported that FEF_25–75%_ was a more numerically sensitive index than FEV_1_ in detecting airway obstruction and response to bronchodilators [4]. However, current data on the value of FEF_25–75%_ compared with FEV_1_ are limited. FEF_25–75%_ has been described as less effort-dependent than FEV_1_ and is a measurement of small airway dysfunction [2, 3]. In a study comparing children aged 10–18 years with normal FEV_1_ (> 80% predicted) and FEF_25–75%_ (> 60% predicted) with those who had normal FEV_1_ (> 80% predicted) and low FEF_25–75%_, reduced FEF_25–75%_ in the presence of normal FEV_1_ was associated with increased asthma severity and reversible airflow obstruction [2, 3]. However, it was noted that there is no guideline regarding normal values for FEF_25–75%_ in children, therefore the authors defined normal FEF_25–75%_ as > 60% predicted, using a value corresponding to 1 standard deviation from the mean FEF_25–75%_ obtained from the initial cohort [3]. A separate study supported this finding proposing FEF_25–75%_ > 65% predicted as normal [5].

Since FEF_25–75%_ correlates well with bronchodilator responsiveness in children with asthma and may reflect peripheral airway obstruction in the presence of a normal FEV_1_ [2, 3], this prompted the evaluation of this parameter in relation to the long-acting muscarinic antagonist bronchodilator tiotropium.

Tiotropium Respimat^®^ (Boehringer Ingelheim International GmbH, Ingelheim am Rhein, Germany) has been shown to improve different measures of lung function in clinical studies with both children and adolescents, including FEF_25–75%_ [6–9]. This is a post hoc analysis of four placebo-controlled trials in children and adolescents with symptomatic asthma who remained uncontrolled despite maintenance therapy (inhaled corticosteroids ± long-acting β_2_-agonist ± leukotriene receptor antagonist, Table 1). We compare the change in trough FEF_25–75%_ and trough FEV_1_ (defined as the pre-dose FEF_25–75%_ or FEV_1_, respectively, measured 24 h post previous drug administration, 10 min prior to the evening dose of usual asthma medication and daily dose of randomized treatment) following treatment with either tiotropium Respimat (5 μg or 2.5 μg) or placebo Respimat.

**Table 1:** Baseline patient demographics and disease characteristics (treated set)

Demographic/characteristic	Symptomatic severe asthma		Symptomatic moderate asthma	
	VivaTinA-asthma^®^ (*N* = 400)	PensieTinA-asthma^®^ (*N* = 392)	CanoTinA-asthma^®^ (*N* = 401)	RubaTinA-asthma^®^ (*N* = 397)
Male, *n* (%)	279 (69.8)	242 (61.7)	264 (65.8)	258 (65.0)
Age, years, median (range)	9.0 (6–11)	14.2 (12–17)	8.9 (6–11)	14.3 (11–17)
Race, *n* (%)				
White	358 (89.5)	371 (94.6)	339 (84.5)	368 (92.7)
Asian	2 (0.5)	10 (2.6)	10 (2.5)	13 (3.3)
Black/African American	5 (1.3)	8 (2.0)	7 (1.7)	14 (3.5)
American Indian/Alaska Native	35 (8.8)	3 (0.8)	45 (11.2)	2 (0.5)
Hawaiian/Pacific Islander	0	0	0	0
Ethnicity, *n* (%)				
Hispanic/Latino	72 (18.0)	68 (17.3)	55 (13.7)	42 (10.6)
Never smoked, *n* (%)	–	392 (100)	–	396 (99.7)
No exposure to second-hand smoke, *n* (%)	369 (92.3)	367 (93.6)	372 (92.8)	353 (88.9)
Age at onset of asthma, years, mean ± SD	4.1 ± 2.4	6.5 ± 3.9	4.7 ± 2.4	6.5 ± 4.1
Duration of asthma, years, median (range)	4.9 (0.6–11.0)	7.8 (0.3–16.5)	4.2 (0.5–11.0)	7.9 (0.3–16.3)
Concomitant diagnoses, *n* (%)				
Allergic rhinitis	238 (59.5)	225 (57.4)	230 (57.4)	219 (55.2)
Atopic dermatitis	38 (9.5)	38 (9.7)	55 (13.7)	37 (9.3)
FEV_1_, l, mean ± SD^a,b^	1.57 ± 0.35	2.53 ± 0.62	1.63 ± 0.39	2.75 ± 0.66
FEV_1_, % predicted, mean ± SD^a,b^	81.64 ± 11.45	79.52 ± 11.49	84.06 ± 10.79	82.79 ± 10.56
FEV_1_% reversibility, median (Q1–Q3)^c,d^	24.03 (17.44–34.10)	26.01 (18.31–36.60)	23.19 (16.94–33.60)	23.29 (17.46–33.76)
FVC, l, mean ± SD^a,b^	2.05 ± 0.48	3.32 ± 0.81	2.12 ± 0.56	3.56 ± 0.86
FVC, % predicted, mean ± SD^a,b^	92.34 ± 13.60	91.62 ± 14.69	94.70 ± 14.71	93.70 ± 13.34
FVC, % reversibility, median (Q1–Q4)^c,d^	12.92 (7.00–22.90)	14.15 (7.35–25.62)	13.62 (6.15–26.35)	12.76 (5.03–25.81)
FEV_1_/FVC ratio, %, mean ± SD^c^	77.36 ± 10.12	76.87 ± 11.26	77.90 ± 10.08	77.89 ± 10.44
FEF_25–75%_, l/second, mean ± SD^a,b^	1.39 ± 0.57	2.23 ± 0.96	1.43 ± 0.58	2.48 ± 0.97
FEF_25–75%_, % predicted, mean ± SD^a,b^	61.30 ± 23.18	61.55 ± 23.06	62.44 ± 22.53	66.09 ± 20.93
FEF_25–75%_, % reversibility, median (Q1–Q4)^c,d^	51.45 (30.60–79.56)	52.61 (29.38–88.79)	48.00 (28.99–79.78)	46.48 (27.12–71.58)
ACQ score, mean ± SD^b,e^	1.966 ± 0.359	2.13 ± 0.43	1.868 ± 0.309	2.03 ± 0.43
Concomitant therapies at baseline, *n* (%)				
LTRAs	339 (84.8)	315 (80.4)	107 (26.7)	33 (8.3)
LABAs	314 (78.5)	324 (82.7)	1 (0.2)	1 (0.3)
ICS dose of stable maintenance treatment (μg; budesonide or equivalent dose), mean ± SD	457.4 ± 236.0	747.0 ± 357.7	310.0 ± 112.0	539.4 ± 292.7

Data from each study includes from all treatment arms

^a^Pre-bronchodilator

^b^Measured at randomization (Visit 2)

^c^Measured at screening (Visit 1)

^d^Reversibility was calculated using measurements of lung function before and 15–30 min after patients inhaled 400 µg salbutamol

^e^ACQ-IA in CanoTinA-asthma^®^ and VivaTinA-asthma^®^

*ACQ* Asthma Control Questionnaire, *ACQ-IA* interviewer-administered ACQ, *FEF*_*25–75%*_ forced expiratory flow at 25–75% of the pulmonary volume, *FEV*_*1*_ forced expiratory volume in 1 s, *FVC* forced vital capacity, *ICS* inhaled corticosteroid, *LABA* long-acting β_2_-agonist, *LTRA* leukotriene receptor antagonist, *SD* standard deviation

We analyzed data from four double-blind, parallel-group, randomized, placebo-controlled phase III trials: VivaTinA-asthma^®^ (NCT01634152; a 12-week trial in 6 to 11-year-old patients with symptomatic severe asthma) [7], PensieTinA-asthma^®^ (NCT01277523; a 12-week trial in 12 to 17-year-old patients with symptomatic severe asthma) [6], CanoTinA-asthma^®^ (NCT01634139; a 48-week trial in 6 to 11-year-old patients with symptomatic moderate asthma) [8] and RubaTinA-asthma^®^ (NCT01257230; a 48-week trial in 12 to 17-year-old patients with symptomatic moderate asthma) [9]. Details for each study have been published previously [6–9]. Each study was conducted in accordance with the amended Declaration of Helsinki. The ethics research boards of the respective institutions approved the protocols, and signed, informed consent was obtained from all patients and/or their parents [6–9].

We compared change from baseline in trough FEV_1_ and trough FEF_25–75%_ at the time of the primary endpoint (PensieTinA- and VivaTinA-asthma at week 12; RubaTinA- and CanoTinA-asthma at week 24), and analyzed correlations between FEF_25–75%_ response and FEV_1_ response at these time points. Pearson’s correlation coefficients were calculated between trough FEV_1_ and trough FEF_25–75%_, pooling data from all treatment arms. As measurement of FEF_25–75%_ relies on accurate measurement of forced vital capacity (FVC), mean FEF_25–75%_ was calculated from the maneuver (≥ 3 and ≤ 8 maneuvers per time point) with the largest total sum of FEV_1_ and FVC. Use and daily calibration of all spirometers used in the four pediatric trials discussed in this analysis met American Thoracic Society/European Respiratory Society criteria [10].

**Fig. 1:**
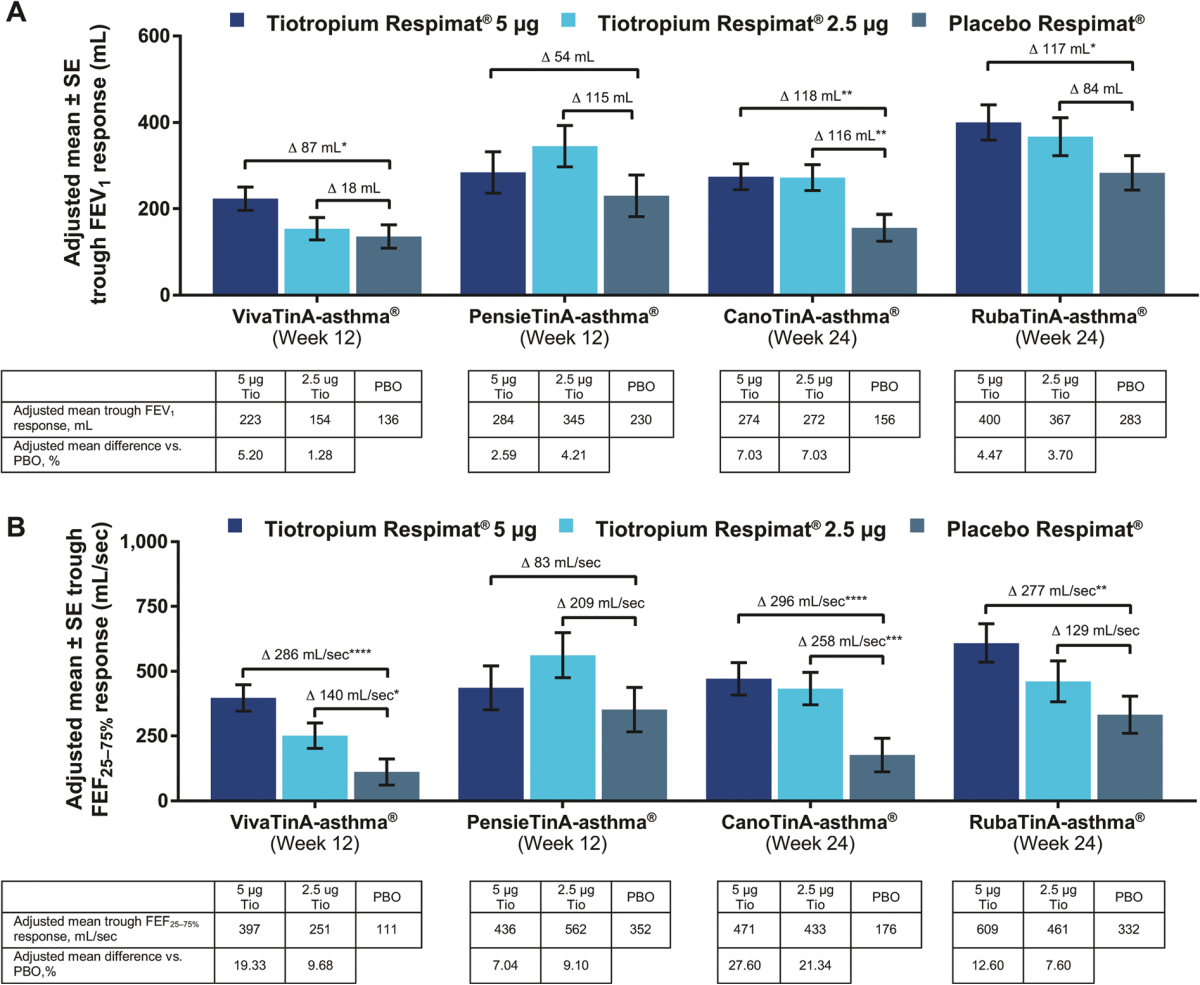
**a** Trough FEV_1_ response; **b** trough FEF_25–75%_ response. *P < 0.05; **P < 0.01; ***P < 0.001; ****P < 0.0001. *FEF*_*25–75%*_ forced expiratory flow at 25–75% of the pulmonary volume, *FEV*_*1*_ forced expiratory volume in 1 s, *PBO* placebo, *SE* standard error, *Tio* tiotropium

A total of 1590 patients were included in the analysis (Table 1). Across the trials in 6 to 11- and 12 to 17-year-old patients with moderate/severe asthma, tiotropium Respimat consistently improved trough FEV_1_ (Fig. 1a) and trough FEF_25–75%_ (Fig. 1b) versus placebo; although in the PensieTinA-asthma study in adolescents with severe asthma, the observed improvements were not statistically significant, possibly due to a pronounced placebo response, which left little room for differentiation between the treatment groups [6].

Improvements in trough FEF_25–75%_ response with tiotropium add-on therapy versus placebo were largely more pronounced than improvements in trough FEV_1_, as evidenced both by the numerical changes and the percentage difference, suggesting that trough FEF_25–75%_ may be a more sensitive measure to detect treatment response, certainly to tiotropium, than trough FEV_1_. Statistical assessment of the correlation between changes in trough FEF_25–75%_ and changes in trough FEV_1_ showed moderate-to-high correlations (0.73–0.80; Supplementary Fig. 1).

Even though assessment of FEF_25–75%_ is not currently recommended in asthma guidelines, there are good arguments for its use to supplement FEV_1_ measurements, particularly in children with asthma. During the early stages of asthma, higher, near-normal FEV_1_ and FEV_1_/ FVC values may obscure airway involvement caused by an inflammatory process, whereas FEF_25–75%_ may signify early functional airway impairment, especially of peripheral airways [11]. FEF_25–75%_ may also better reflect small airways disease due to peripheral positioning of the airflow choke point in patients with mid-to-low lung volumes [2]. Compared with FEV_1_, low FEF_25–75%_ may be a more sensitive indicator of childhood symptomatic asthma, whereas FEV_1_ in children can be normal, even in the presence of symptoms of uncontrolled asthma [3]. Indeed, in our study of children with symptomatic moderate or severe asthma, mean FEV_1_ percent predicted at baseline was predominantly in or just under the normal range (≥ 80%). It has been suggested that FEF_25–75%_ may be a functional marker of asthma severity, whereby low FEF_25–75%_ alongside normal FEV_1_ is associated with increased asthma severity, systemic steroid use, and asthma exacerbations in children [3].

Furthermore, FEF_25–75%_ has been shown to correlate well with bronchodilator (short-acting β_2_-agonist) responsiveness in children with asthma who have normal FEV_1_ [2], and it may therefore be a helpful measure to predict which patients might benefit from further bronchodilation [2]. Certainly, in our study, FEF_25–75%_ was useful in detecting treatment response to tiotropium. However, it should be noted that tiotropium, as a bronchodilator, may have other mechanisms of action affecting small as well as large airways, although there is currently no evidence of this. A previous study using a different bronchodilator, albuterol, with an alternative delivery device (pressurized metered dose inhaler), provided results in accordance with those reported here, further supporting the utility of FEF_25–75%_ as a more sensitive measure of airway response to bronchodilators than FEV_1_ in children and adolescents with asthma, irrespective of delivery device [4].

FEF_25–75%_ has certain limitations, including a larger variability than FEV_1_, particularly in adults, and its reliance on the valid measurement of FVC [12, 13]. As FVC and total lung capacity can be affected by disease progression or therapeutic interventions, FEF_25–75%_ pre- and post-interventions may be not be comparable [13]. Ideally, measurements should be standardized for total lung capacity, but this is not usually feasible and the vital capacity is used as a proxy for lung size [13], and was not possible within this post hoc analysis. The potential lack of specificity means that FEF_25–75%_ by itself may have limited diagnostic value. Quanjer et al. previously challenged both the usefulness of FEF_25–75%_ as a clinical marker and the hypothesis that reduced mid-expiratory flows are specific for small airways disease [13]. Yet, this analysis provides further support to the literature that suggests the use of FEF_25–75%_ may help in the identification of children and adolescents who may have a normal FEV_1_ but significant asthma symptoms, or who may require further evaluation from a healthcare professional or adjustments to their treatment regimen [14]. In addition, since this analysis of four studies is probably the largest to investigate the effect of a bronchodilator on FEF_25–75%_ in children and adolescents with asthma, the suggestion that FEF_25–75%_ should be used as an additional lung function measurement is appropriate.

In conclusion, our results strengthen the evidence that FEF_25–75%_ should be evaluated as an additional lung function measurement in pediatric patients. Moreover, FEF_25–75%_ may contribute as a measure to detect response to treatment.

## Electronic Supplementary Material

Below is the link to the electronic supplementary material.

Supplementary Figure 1. Correlation between trough FEV_1_ and trough FEF_25–75%_ response. A. VivaTinA-asthma^®^; B. PensieTinA asthma^®^; C. CanoTinA-asthma^®^; D. RubaTinA-asthma^®^. PCC: <0.2, very weak correlation; 0.2–0.4, low correlation; 0.4–0.75, moderate correlation; 0.75–0.9, high and substantial correlation; 0.9–1.0, very high/certain correlation. Abbreviations: FEF_25–75%_, forced expiratory flow at 25–75% of the pulmonary volume; FEV_1_, forced expiratory volume in 1 second; PCC, Pearson’s correlation coefficient (PDF 317 kb)
