# Supplementary material for: Forced Expiratory Flow (FEF25–75%) as a Clinical Endpoint in Children and Adolescents with Symptomatic Asthma Receiving Tiotropium: A Post Hoc Analysis
Source: Pulm Ther. 2020 May 12;6(2):151–8. doi: 10.1007/s41030-020-00117-6 (PMC7672130; doi:10.1007/s41030-020-00117-6)
Supplement: Supplementary file 1 — Supplementary Figure 1. Correlation between trough FEV1 and trough FEF25–75% response. A. VivaTinA-asthma®; B. PensieTinA asthma®; C. CanoTinA-asthma®; D. RubaTinA-asthma®. PCC: <0.2, very weak correlation; 0.2–0.4, low correlation; 0.4–0.75, moderate correlation; 0.75–0.9, high and substantial correlation; 0.9–1.0, very high/certain correlation. Abbreviations: FEF25–75%, forced expiratory flow at 25–75% of the pulmonary volume; FEV1, forced expiratory volume in 1 second; PCC, Pearson’s correlation coefficient (PDF 317 kb) [file 41030_2020_117_MOESM1_ESM.pdf]

Supplementary Figure 1. Correlation between trough FEV_1 and trough $FEF_{25–75\%}$ response.
A. VivaTinA-asthma®; **B.** PensieTinA asthma®; **C.** CanoTinA-asthma®; **D.** RubaTinA-asthma®

A.

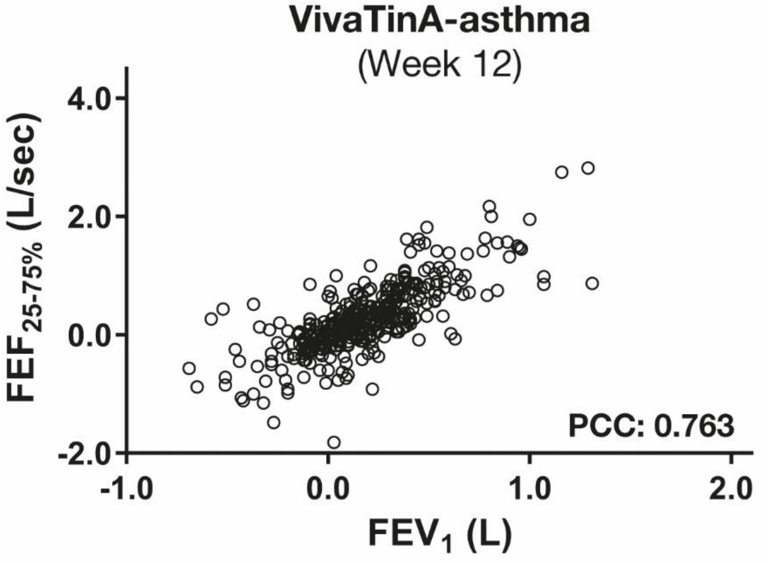

B.

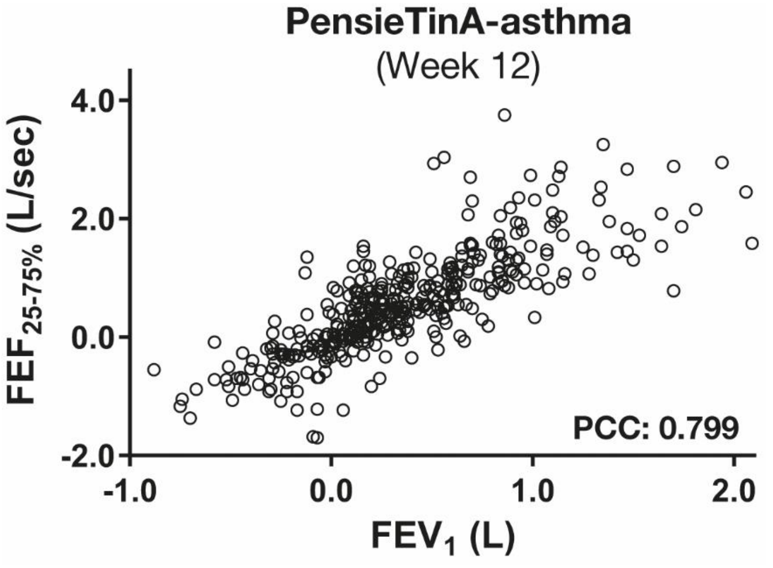

C.

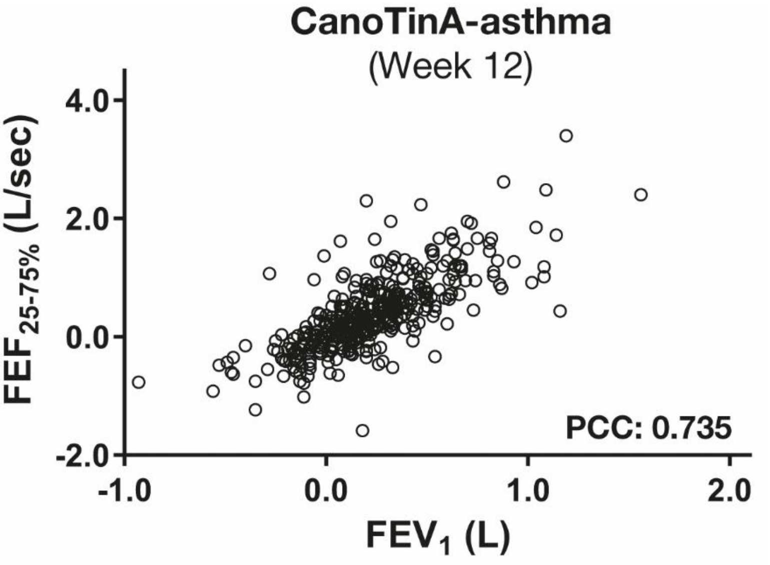

D.

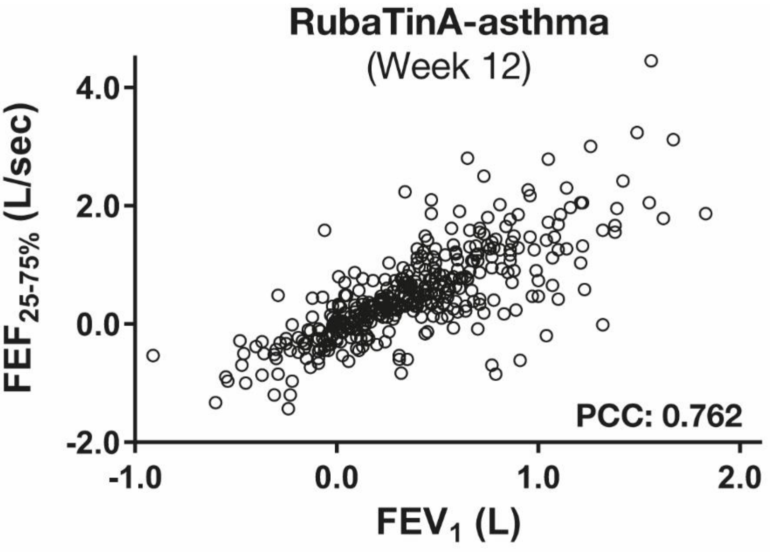

PCC: <0.2, very weak correlation; 0.2–0.4, low correlation; 0.4–0.75, moderate correlation; 0.75–0.9, high and substantial correlation; 0.9–1.0, very high/certain correlation.

Abbreviations: $FEF_{25–75\%}$, forced expiratory flow at 25–75% of the pulmonary volume; FEV_1, forced expiratory volume in 1 second; PCC, Pearson's correlation coefficient.
